# Evaluation of morbidity in *Schistosoma mansoni*-positive primary and secondary school children after four years of mass drug administration of praziquantel in western Kenya

**DOI:** 10.1186/s40249-020-00690-7

**Published:** 2020-06-15

**Authors:** Bernard O. Abudho, Bernard Guyah, Bartholomew N. Ondigo, Eric M. Ndombi, Edmund Ireri, Jennifer M. Carter, Diana K. Riner, Nupur Kittur, Diana M. S. Karanja, Daniel G. Colley

**Affiliations:** 1 Centre for Global Health Research (KEMRI-CGHR), Kenya Medical Research Institute, Kisumu, Kenya; 2 Department of Biomedical Sciences and Technology, Maseno University, Maseno, Kenya; 3 Department of Biochemistry and Molecular Biology, Egerton University, Nakuru, Kenya; 4 Laboratory of Malaria Immunology and Vaccinology, National Institute of Allergy and Infectious Diseases, NIH, Bethesda, MD USA; 5 Department of Pathology, Kenyatta University, Nairobi, Kenya; 6 Centre for Clinical Research-Radiology Unit, Kenya Medical Research Institute, Nairobi, Kenya; 7 Center for Tropical and Emerging Global Diseases, University of Georgia, Athens, GA USA; 8 Department of Microbiology, University of Georgia, Athens, GA USA

**Keywords:** Schistosomiasis, Kenya, Mass drug administration, School-based, Morbidity, School pediatrics quality of life scores

## Abstract

**Background:**

World Health Organization guidelines recommend preventive chemotherapy with praziquantel to control morbidity due to schistosomiasis. The primary aim of this cross-sectional study was to determine if 4 years of annual mass drug administration (MDA) in primary and secondary schools lowered potential markers of morbidity in infected children 1 year after the final MDA compared to infected children prior to initial MDA intervention.

**Methods:**

Between 2012 and 2016 all students in two primary and three secondary schools within three kilometers of Lake Victoria in western Kenya received annual mass praziquantel administration. To evaluate potential changes in morbidity we measured height, weight, mid-upper arm circumference, hemoglobin levels, abdominal ultrasound, and quality of life in children in these schools. This study compared two cross-sectional samples of *Schistosoma mansoni* egg-positive children: one at baseline and one at year five, 1 year after the fourth annual MDA. Data were analyzed for all ages (6–18 years old) and stratified by primary (6–12 years old) and secondary (12–18 years old) school groups.

**Results:**

The prevalence of multiple potential morbidity markers did not differ significantly between the egg-positive participants at baseline and those at 5 years by Mann Whitney nonparametric analysis and Fisher’s exact test for continuous and categorical data, respectively. There was a small but significantly higher score in school-related quality of life assessment by year five compared to baseline by Mann Whitney analysis (*P* = 0.048) in 13–18 year olds where malaria-negative. However, anemia was not positively impacted by four annual rounds of MDA, but registered a significant negative outcome.

**Conclusions:**

We did not detect differences in morbidity markers measured in a population of those infected or re-infected after multiple MDA. This could have been due to their relative insensitivity or a failure of MDA to prevent morbidity among those who remain infected. High malaria transmission in this area and/or a lack of suitable methods to measure the more subtle functional morbidities caused by schistosomiasis could be a factor. Further research is needed to identify and develop well-defined, easily quantifiable *S. mansoni* morbidity markers for this age group.

## Background

Human schistosomiasis caused by the trematode *Schistosoma mansoni* is endemic in western Kenya near Lake Victoria where its prevalence has an inverse relationship with the distance from the shore [1, 2]. The Passage of World Health Assembly (WHA) Resolution 54.19 in 2001 led to renewed emphasis on reducing the global burden of schistosomiasis. The current World Health Organization (WHO) strategy to reduce schistosomiasis-associated morbidity is mass drug administration (MDA) with praziquantel (PZQ) based on the prevalence and intensity of infection in an area [3]. Most commonly this translates to yearly or biennial MDA with PZQ in primary schools to treat schistosomiasis in addition to administration of albendazole (ALB) for soil-transmitted helminths (STHs) [4]. The effectiveness of this strategy is usually monitored by MDA coverage and by changes in prevalence and/or intensity of infection rather than determination of changes in morbidity [4–9].

While the goal of PZQ MDA is to control morbidity, because many of the sequelae associated with schistosomiasis can have other causes, it has proven challenging to easily measure morbidity specifically resulting from schistosomiasis. Pathology due to *S. mansoni* infection is caused largely by chronic deposition of parasite eggs in the liver and intestines, which results in egg-focused granuloma formation and focal and systemic inflammation [5, 10]. This, in turn leads to sequelae that can range from “subtle or functional morbidities” such as anemia, physical (growth stunting, wasting) and cognitive disabilities, which can impact a child’s quality of life [11–13], to periportal fibrosis, portal hypertension, hematemesis, and death. While severe disease likely develops in only 5 to 10% of those with substantial, untreated chronic infections [14, 15], the subtle morbidities are thought to have a broader public health impact on most of the over 240 million people with schistosomiasis [12, 16, 17]. However, they are more difficult to assess for example specific morbidity assessment of anemia in *S. mansoni* infection is challenging because in many areas where individuals are at risk for schistosomiasis, they are also at risk for malaria and both infections cause anemia. The synergistic effect of co-infection and potential interaction outcome of schistosomiasis and malaria infections on anemia may affect the usefulness of anemia as a marker of schistosomiasis [18, 19].

Assessments of morbidity markers associated with *S. mansoni* infection were conducted in two populations; one group prior to MDA (at baseline) and a second group, all that were *S. mansoni* egg-positive 1 year after four rounds of high coverage, school based annual MDA in year five. The population in year five remained *S. mansoni* egg-positive either due to not being cured during the MDA or due to re-infection during the year following the last MDA. Secondary school children are not routinely included in most morbidity assessment studies, so there are limited data for this age group even though they tend to have the highest prevalence and intensity of schistosome infections [20]. Although rapid re-infection can occur after treatment, some data suggest that having received treatment in childhood can reduce morbidity associated with urinary schistosomiasis later in life [21]. The primary aim of this cross sectional study was to address whether 4 years of effective primary and secondary school MDA is enough to lower morbidity in individuals with infections a year after the final MDA compared to children who were *S. mansoni*-positive prior to the initial MDA intervention.

A secondary aim of this cross-sectional study was to evaluate the utility of several potential markers of morbidity to detect changes before and post-MDA in primary and secondary school children. This analysis was done in conjunction with our studies documenting the effectiveness of the four rounds of annual MDA in significantly lowering the prevalence and intensity of *S. mansoni* infection [22] and evaluating immunological outcomes in the same groups of children [23].

## Methods

### Ethics statement

Ethical approval to conduct the study was obtained from the Departmental and Institutional Scientific Steering Committees of Kenya Medical Research Institute (KEMRI) followed by the National KEMRI Scientific and Ethical Review Unit (SERU). The Institutional Review Board (IRB) of the University of Georgia also reviewed and approved the study protocol (ID#00003501). Before the start of the study, informational meetings were held with the teachers, children and parents of the children in the five schools in the study. In these meetings, individual informed permission was first obtained from the parents or guardians of school-going children from Grades 1 to 12; assent to participate in the study was also obtained from each participating child [22].

### Study area and population

This study was conducted between the year 2012 and 2016 in two primary and three secondary schools in fishing villages located within three kilometers of Lake Victoria in the Asembo Bay area of Rarieda Sub-County, Nyanza Region in western Kenya. The target population included all male and female 6 to 18–year-old children attending the five selected schools. The selected primary schools are the main feeder schools for the selected secondary schools [22], and all were selected based on their expected high prevalence of *S. mansoni* infection based on previous studies in the area [1, 7]. Approximately 96% of the population in this area is of the Luo ethnic group and the major occupations are fishing and subsistence farming [24]. The area is highly endemic for *S. mansoni* as reported by a 2001 survey in primary schools that found prevalence ranging from 35 to 85% [1]. More recent findings [7, 25] also support high rates of schistosomiasis in the region.

### Study design

Parasitologic data were collected at baseline and morbidity testing was conducted in those that were egg positive. Annual MDA with PZQ and albendazole was provided to all children attending the five selected schools PZQ (40 mg/kg) and ALB (400 mg). This was done by directly observed therapy conducted by trained members of the study team. Because there was very high school attendance (> 90%) and extensive promotion of the MDAs, treatment coverage was > 90% [22]. Malaria positive children were treated with artemether-lumefantrine. An annual parasitologic survey was conducted in a randomly selected sub-set of children from all grades in each of the five schools prior to each annual MDA, using a repeated cross sectional study design [22]. Morbidity data from a cross section of children who were egg positive at year five, 1 year after the last of four annual school-wide MDAs, were compared to data from the cross section of children of similar age group at baseline.

### Parasitologic surveys

Schistosomiasis and STHs were diagnosed as previously described [22]. Briefly, children were enrolled and asked to provide fresh stool samples for *S. mansoni* and STH infection assessments by the Kato-Katz technique [26]. One stool sample was collected at baseline and three stool samples were collected in subsequent years; two Kato-Katz slides were prepared per stool and examined for parasite eggs by two independent, well-trained and experienced microscopists. Examination of slides for hookworm eggs were performed within 1 h of slide preparation and the presence of STH eggs (hookworms, *Ascaris lumbricoides* and *Trichuris trichiura*) were recorded as either positive or negative. Quantitative counts of eggs were performed only for *S. mansoni*. The intensity of *S. mansoni* infection was expressed as eggs per gram of feces (EPG) by multiplying the arithmetic mean of egg counts from the total slides per child by 24 and categorized according to WHO guidelines as light (1–99 EPG), moderate (100–399 EPG) and heavy (≥ 400 EPG) [3, 9].

### Blood collection and processing

Approximately 100 μl of blood was collected and tested for hemoglobin levels using a portable, battery operated hemoglobinometer (Hemocue®, Angelholm, Sweden) and expressed in g/dl then converted to g/L by dividing the measurement by 10. Anemia was defined according to Kenya National guidelines [27]. Malaria infection status was determined by examining a Giemsa stained blood smear for malaria parasite and quantified by the number of infected red blood cells per 300 leukocytes. Positive infection status was defined as the presence of a single *Plasmodium falciparum* parasite.

### Ultrasonographic examination

A portable ultrasound machine (SSD-500 Aloka, Tokyo, Japan) equipped with 3.5 MHz convex abdominal probe was used to examine study participants for hepatosplenic and portosystemic morbidity according to WHO Niamey protocol guidelines [28]. Ultrasonography was performed by a highly experienced senior radiographer (E.I.) familiar with the WHO Niamey protocol. Ultrasound assessments were graded as image patterns A to F; A and B were considered normal. Splenomegaly (SM), hepatomegaly (HM), portal branch thickening (PBT) and increased portal vein diameter (PVDs) were defined as values 2SDs above a schistosome-free Senegalese reference population after adjusting for age and height [29].

### Pediatrics Quality of Life (PedsQoL) measurements

For quality of life estimates, the PedsQ™ 4.0 Generic Core Scales child self-report questionnaire was used. The instrument is designed for ages 5–18 and consists of 23 items grouped into 4 domains: Physical Functioning (PF), Emotional Functioning (EF), Social Functioning (SF) and School Functioning (SchF) comprising of 8, 5, 5 and 5 items, respectively. Details are available at https://www.pedsql.org/about_pedsql.html. The survey questions assessed how much of a problem each item has been during the past month. A 5-point Likert-like response scale is used and scored as: 0 (never a problem), 1 (almost never), 2 (sometimes), 3 (often) and 4 (almost always a problem). Responses were linearly transformed to 100, 75, 50, 25 and 0, respectively, resulting to a 0–100 scale (0 = 100, 1 = 75, 2 = 50, 3 = 25, 4 = 0) with higher scores representing better level of functioning and better QoL. Parental proxy reports were not collected.

The PedsQoL overall and sub-scale scores were computed as the sum of the items divided by the number of items answered (accounting for the missing data). Because we had very few missing data, this allowed for all scale scores to be computed. If 50% of the items in the subscale are missing, the scale score is not computed. Physical Health Summary score (8 items) is the same as the Physical functioning subscale. The Psychosocial Health summary score (15 items) is computed as the sum of the items divided by the number of the items answered in the emotional, social, and school functioning subscales. Total scores are also presented [30].

Before the initiation of the study, The PedsQ™ 4.0 was translated into Dholuo and then back translated into English (Dholuo-English). The approved Dholuo version was first pre-tested on 30 randomly selected children, 15 each in the 6–12 year (primary school) and the 13–18 year (secondary school) age groups. These children were not reassessed in the actual QoL survey, but they were in the study schools. All the PedsQ™ 4.0 questionnaires in this study were administered by well-trained research assistants who are native Dholuo speakers.

### Data management and analyses

The location of each school was determined using a Global Positioning System on Android phones. Study participants were given unique identification numbers and all data were entered using Microsoft Excel version 14.0 (Microsoft Corporation, Redmond, Washington, USA) and kept confidential. Data were subsequently analyzed using IBM SPSS version 24 (IBM Corporation, Armank, New York, USA) and GraphPad Prism version 6 (GraphPad Software, La Jolla, California, USA). We compared data from two cross sectional surveys of children. Mann Whitney nonparametric analysis and Fisher’s exact test were used for evaluating differences between years for continuous data and categorical data, respectively. Tests were considered statistically significant at *P* <  0.05.

## Results

### Demographics of the *S. mansoni*-positive study groups

A total of 295 *S. mansoni*-positive school-attending children were enrolled in the cross sectional baseline assessment; 51.9% were female. One year after four rounds of annual MDA, there were 69 individuals who were *S. mansoni* egg-positive and were therefore included in the year five cohort; 47.8% were female. The median age was 13 years both in the baseline group and in the year five group. There were 117 *S. mansoni* egg positive children in 6–12 years old in the baseline group and 32 in the year 5 group. For 13–18 years old, there were 178 *S. mansoni* egg positive in the baseline group and 37 in the year five group (Table 1). The smaller number of egg-positive participants available at year five and the lower intensities of infection among these participants compared to the baseline cohort (Table 1).

**Table 1:** Demographics and prevalence of other parasitic diseases in *Schistosoma mansoni-*positive children by age group at baseline and year five

	*Schistosoma mansoni* positive		
	Baseline	Year five	*P*-value
**All ages**	*n* = 295	*n* = 69	
Schistosomiasis prevalence	295/295 (100%)	69/368 (18.8%)	0.000^*^
Median age (years)	13 (6–18)	13 (7–18)	0.180
Female *n* (%)	153 (51.9)	33 (47.8)	0.594
Malaria *n* (%)	118 (40.0)	31 (44.9)	0.494
EPG – Median (range)	80 (30–1746)	12 (4–366)	0.000^*^
Any STH	66 (22.4)	7 (10.1)	0.029^*^
*Ascaris lumbricoides*	23 (7.80)	2 (2.90)	0.277
*Trichuris trichiura*	32 (10.9)	5 (7.3)	0.508
Hookworm	21 (7.1)	0	0.019^*^
**6–12 years old (Primary school)**	*n* = 117	*n* = 32	
Median age (years)	11 (6–12)	10 (7–12)	0.502
Female *n* (%)	63 (53.9)	14 (43.8)	0.326
Malaria *n* (%)	60 (51.3)	18 (56.3)	0.691
EPG – Median (range)	96 (6–1746)	16 (4–132)	0.000^*^
Any STH	33 (28.1)	6 (18.8)	0.366
*A. lumbricoides*	11 (9.40)	2 (6.3)	0.735
*T. trichiura*	18 (15.4)	4 (12.5)	0.786
Hookworm	8 (6.84)	0	0.202
**13–18 years old (Secondary school)**	*n* = 178	*n* = 37	
Median age (years)	16 (13–18)	15 (13–18)	0.509
Female *n* (%)	90 (50.6)	19 (51.4)	0.999
Malaria *n* (%)	58 (32.6)	13 (35.1)	0.848
EPG – Median (range)	74 (4–1284)	8 (4–366)	0.000^*^
Any STH	33 (18.5)	1 (2.70)	0.013^*^
*A. lumbricoides*	12 (6.74)	0	0.228
*T. trichiura*	14 (7.87)	1 (2.70)	0.477
Hookworm	13 (7.30)	0	0.131

Differences among the study groups were evaluated using Mann-Whitney U test for continuous data and Fisher’s exact test for categorical data

*Abbreviations*: *EPG* Eggs per gram of feces, *STH* Soil transmitted helminths

An asterisk (^*^) indicates statistical significance 1 year after four rounds of annual MDA with praziquantel

### *S. mansoni,* soil transmitted helminth, and malaria infections in baseline and year five groups

The overall *S. mansoni* prevalence in 6–18 years old at baseline and in the year five group was 100 and 18.75% respectively (Table 1). The median egg burden was 80 EPG in the baseline group and 12 EPG in the year 5 group (*P* < 0.001). The overall prevalence of any STH was 22.4% (*n* = 66) in the baseline survey group and 7% (*n* = 10) in the year five group (*P* = 0.029). With regard to individual STH infections, hookworm infection was 7.1% (*n* = 21) in the baseline group and 0% in the year five group (*P* = 0.019). The prevalence of *Trichuris trichiura* was 10.9% (*n* = 32) in the baseline group and 7.3% (*n* = 5) in the year five group (*P* = 0.508). And *Ascaris lumbricoides* infection prevalence was 7.8% (*n* = 23) in the baseline group and 2.9% (*n* = 2) in the year five group (*P* = 0.277). The overall point prevalence of malaria was 40% (*n* = 118) and 44.9% (*n* = 31) in the baseline and year five groups respectively (*P* = 0.494).

### Prevalence of anemia in *S. mansoni* positive children in baseline and year five groups

Anemia prevalence was significantly higher in *S. mansoni* positive children in the year five group than that of baseline group prior to MDA (*P* <  0.001, Table 2). Statistical differences remained when age groups were separated into 6–12 years old and 13–18 years old. It did not appear to be a consequence of differences in malaria infections as the prevalence of anemia was similarly high in both malaria positive and malaria negative individuals in the 5 year group than in baseline group.

**Table 2:** Prevalence of anemia in *Schistosoma mansoni*-positive children in baseline and year five groups

	Baseline		Year five		
	*n*	Anemic (%)	*n*	Anemic (%)	*P*-value
**All ages**	295	73 (24.8)	69	47 (68.1)	< 0.001
Malaria positive	118	36 (30.5)	31	19 (61.3)	0.003
Malaria negative	177	37 (20.9)	38	28 (73.7)	< 0.001
**6–12 years old (Primary school)**	117	38 (32.5)	32	18 (56.2)	0.022
Malaria positive	60	22 (36.7)	18	10 (55.6)	0.179
Malaria negative	57	16 (28.1)	14	8 (57.1)	0.058
**13–18 years old (Secondary school)**	178	35 (19.7)	37	29 (78.4)	< 0.001
Malaria positive	58	14 (24.1)	13	9 (69.2)	0.003
Malaria negative	120	21 (17.5)	24	20 (83.3)	< 0.001

Differences between cross-sectional groups were evaluated using Fisher’s exact test

### Comparison of quality of life scores

There were no differences in median scores for the physical (PedsQoL-physical), emotional (PedsQoL-emotional), social (PedsQoL-social) and total (PedsQoL-total) quality of life scores children in the overall group (6–18 years old) between the baseline and year five groups. There was a small but statistically significant (*P* = 0.048) increase (i.e., QoL improvement) in median scores for the school PedsQoL in the malaria negative secondary school (13–18 years old groups) between baseline and year five (Table 3). None of the median PedsQoL scores for (6–12 years old group) differed significantly between baseline and year five groups (data not shown).

**Table 3:** PedsQoL data according to malaria infection in *Schistosoma mansoni*-positive secondary school children at baseline and year five

	Baseline Median (range)	Year five Median (range)	*P*-value
All 13–18 years old	*n* = 182	*n* = 37	
PedsQoL-Physical	81.3 (12.5–100)	78.1 (53.1–100)	0.599
PedsQoL-Emotional	67.5 (30.0–100)	70.0 (40.0–100)	0.218
PedsQoL-Social	80.0 (35.0–100)	80.0 (45.0–100)	0.821
PedsQoL-School	70.0 (10.0–100)	75.0 (55.0–100)	0.021
PedsQoL-Total	75.0 (39.1–96.7)	77.1 (55.4–98.9)	0.344
**13–18 years old malaria positive**	*n* = 58	*n* = 13	*P*-value
PedsQoL-Physical	79.7 (68.8–90.6)	78.1 (78.1–84.4)	0.929
PedsQoL-Emotional	70.0 (55.0–80.0)	70.0 (60.0–75.0)	0.952
PedsQoL-Social	80.0 (70.0–90.0)	85.0 (70.0–90.0)	0.875
PedsQoL-School	70.0 (60.0–90.0)	80.0 (70.0–85.0)	0.321
PedsQoL-Total	77.2 (65.2–85.9)	79.3 (68.5–84.8)	0.572
**13–18 years old malaria negative**	*n* = 124	*n* = 24	*P*-value
PedsQoL-Physical	81.3 (67.2–90.6)	76.6 (68.8–84.4)	0.472
PedsQoL-Emotional	65.0 (55.0–80.0)	77.5 (57.5–85.0)	0.211
PedsQoL-Social	80.0 (70.0–90.0)	77.5 (65.0–92.5)	0.823
PedsQoL-School	70.0 (60.0–80.0)	75.0 (70.0–85.0)	0.048^*^
PedsQoL-Total	73.9 (63.6–83.7)	74.5 (67.9–81.5)	0.491

Differences among the study groups were evaluated using Mann-Whitney U test

An asterisk (^*^) indicates statistical significance between the groups 1 year after four rounds of annual MDA with praziquantel

### Prevalence of organomegaly, wasting and stunting

The overall prevalence of organomegaly, stunting and wasting was similar in the baseline and year five groups (Tables 4 and 5). A smaller proportion of children had hepatomegaly or hepatosplenomegaly in the year five group than in the baseline group but these differences were not statistically significant. By contrast, there was a higher proportion of children with splenomegaly in the year five group compared to baseline group but it also was not a significant difference. Stratifying the groups based on malaria status, which can also affect liver and spleen pathology, did not reveal any statistically significant differences. No children in either age group had evidence of schistosomiasis-associated fibrosis (image pattern ≥ C) by ultrasound at either time point. Because fibrotic image pattern was rare in both groups, evaluating statistical significance was not warranted.

**Table 4:** Organomegaly, wasting and stunting prevalence in *Schistosoma mansoni*-positive primary school children at baseline and year five

	*Schistosoma mansoni* positive, *n* (%)		
	Baseline	Year five	*P*-value
**All 6–12 years old**	*n* = 117	*n* = 32	
Hepatomegaly alone	26 (22.2)	6 (18.8)	0.810
Hepatosplenomegaly	36 (30.8)	9 (28.1)	0.831
Splenomegaly alone	25 (21.4)	10 (31.3)	0.248
Normal	30 (25.6)	7 (21.9)	0.818
Stunting	5 (4.4)	0	0.585
Wasting	1 (0.9)	0	0.999
**6–12 years old malaria positive**	*n* = 60	*n* = 18	
Hepatomegaly	11 (18.3)	0	0.059
Hepatosplenomegaly	23 (38.3)	7 (38.9)	0.999
Splenomegaly	15 (25.0)	8 (44.4)	0.143
Normal	11 (18.3)	3 (16.7)	0.999
Stunting	3 (5.0)	0	0.999
Wasting	1 (1.7)	0 (0.0)	0.999
**6–12 years old malaria negative**	*n* = 57	*n* = 14	
Hepatomegaly	15 (26.3)	6 (42.9)	0.326
Hepatosplenomegaly	13 (22.8)	2 (14.3)	0.719
Splenomegaly	10 (17.5)	2 (14.3)	0.999
Normal	19 (33.3)	4 (28.6)	0.999
Stunting	2 (3.5)	0	0.999
Wasting	0	0	0.999

Differences among the two study groups were evaluated using Fisher’s exact test

**Table 5:** Organomegaly, wasting and stunting in older *Schistosoma mansoni*-positive secondary school children at baseline and year five

	Baseline	Year five	*P*-value
**All 13–18 year old**	*n* = 178 (%)	*n* = 37 (%)	
Hepatomegaly alone	24 (13.5)	4 (10.8)	0.793
Hepatosplenomegaly	31 (17.4)	4 (10.8)	0.463
Splenomegaly alone	33 (18.5)	6 (16.2)	0.819
Normal	90 (50.6)	23 (62.2)	0.211
Stunting	8 (4.5)	2 (5.4)	0.683
Wasting	1 (0.6)	2 (5.4)	0.077
**13–18 years old malaria positive**	*n* = 58 (%)	*n* = 13 (%)	
Hepatomegaly	6 (10.3)	2 (15.4)	0.633
Hepatosplenomegaly	15 (25.9)	2 (15.4)	0.720
Splenomegaly	18 (31.0)	1 (7.7)	0.162
Normal	19 (32.8)	8 (61.5)	0.065
Stunting	3 (5.2)	1 (7.7)	0.563
Wasting	0 (0.0)	0 (0.0)	0.999
**13–18 years old malaria negative**	*n* = 120 (%)	*n* = 24 (%)	
Hepatomegaly	18 (15.0)	2 (8.33)	0.528
Hepatosplenomegaly	16 (13.3)	2 (8.3)	0.738
Splenomegaly	15 (12.5)	5 (20.8)	0.330
Normal	71 (59.2)	15 (62.5)	0.823
Stunting	5 (4.2)	1 (4.2)	0.999
Wasting	1 (0.8)	2 (8.3)	0.072

Differences among the two study groups were evaluated using Fisher’s exact test

## Discussion

Although the WHO goal for schistosomiasis control programs is to reduce morbidity, programs are mainly monitored by level of MDA coverage and/or changes in prevalence and intensity of infection. Infection intensity, while not a direct indicator of morbidity, has been associated with severe disease consequences. Because it is relatively easy to quantify in highly endemic areas, it has been used as a proxy for severe disease morbidity [31]. However, because the relationship between intensity of infection and functional morbidity is not direct [32], there is a need for measurements of morbidity that can be readily incorporated into control programs [33].

In this current study, the sample size in year five group was determined by the number of children who were *S. mansoni* egg-positive at year five, a year after the final MDA of the larger, primary study [22]. The primary study was quite successful in controlling the prevalence of schistosomiasis in these five schools through four annual rounds of MDA with PZQ, the resulting sample size of those who were *S. mansoni* egg positive by year five was considerably smaller than that at baseline. This significantly reduced number of individuals who were still *S. mansoni* egg positive in year five constituted the entire group studied in regard to morbidity in this current study.

In this study, we hypothesized that multiple annual rounds of MDA would result in less morbidity, even in children who have *S. mansoni* infection 1 year following the last MDA. However, most of the measures we evaluated were not significantly different between the two time points. In addition, for some traditional markers of schistosomiasis-associated morbidity, such as image pattern detected by ultrasound, the low level of measurable morbidity at baseline prevented the ability to detect any appreciable differences following MDA. In contrast, in year five, anemia which was one of multiple markers evaluated was not positively impacted by four rounds of MDA with praziquantel and registered a significant negative outcome. This could be an indication that this potential marker may not always be a reliable indicator of decreasing prevalence and intensity in a population of school children. Furthermore, others factors such as malaria which was seen to increase in this area during this timeframe [22] could have played a role in the significant increase of anemia in year five despite four rounds of MDA.

Our inability to demonstrate benefits of MDA using the proposed morbidity markers we studied could be attributed to many factors. The bleakest interpretation of our data would be that multiple rounds of MDA did not appreciably reduce the morbidity in school age children who become re-infected. Although infection intensity was lower in the group we surveyed after 4 years of MDA, infected individuals still have inflammation-inducing schistosome eggs in their tissues and tested positive by Kato-Katz. It may be that it is necessary to be egg free to be morbidity free. Alternatively, there may be other measures of morbidity that would have been able to demonstrate differences more clearly.

The principal limitation of our study is that we do not know the characteristics of the year five group prior to MDA. We assumed that they had the similar characteristics to the baseline group but we do not know that. In addition, the 5 year time difference between morbidity measurements could have also reduced our ability to detect differences if events that affect health had occurred in the interim.

Measuring morbidity due to schistosomiasis mansoni in children has proven challenging [33]. This is especially true in areas endemic for malaria [34, 35]. Although there have been malaria control efforts in the study area [24], malaria point prevalence was high (40.0% at baseline and 44.9% at year five) during our study period. Both baseline and year five data collections were done in the same season to mitigate seasonal variation in malaria prevalence.

Because the relationship between malaria and anemia in this area is well documented [35, 36], we analyzed the data together as well as separating individuals based on malaria status but still did not find appreciable differences in the proposed measures of morbidity following repeated MDA. We propose that the high prevalence of malaria seen in our study population may have masked some of the benefits of MDA. In a western Kenya study of primary school students where malaria prevalence was lower (< 15%), significant decreases in wasting and ultrasound-detected organomegaly, along with significantly improved pediatric quality-of-life scores were observed over a 5 year period in children that received either 2 or 4 rounds of MDA [37]. Additional research is needed to identify markers of schistosomiasis morbidity that can be used to monitor the effectiveness of control programs regardless of the underlying malaria prevalence and this could include measurement of inflammatory markers and others such as morbidity markers such as cognitive development, school attendance and behavior, shuttle run test and ascites in subsequent studies [38]. This study was the first to measure potential morbidity markers after population control of *S. mansoni* infection using a school-based MDA among infected children across all primary and secondary school years*.* Most such studies have only evaluated outcomes in primary school children. The high level of infection in secondary school-aged children seen here argues that they should be included in MDA intervention programs and morbidity assessments.

## Conclusions

We failed to find an impact of four rounds of MDA on potential markers of morbidity in primary and secondary school children in western Kenya who were *S. mansoni*-positive at the end of the study. This may either be due to the tested measures not being sensitive markers for morbidity in children, confounding by malaria or other conditions, or the discouraging prospect that there is no lasting benefit of previous MDA for children with schistosome (re)infection. By contrast, comparisons of morbidity between children who have active infection and those that are egg free demonstrate clear differences. This suggests that while the goal of most intervention programs is to control morbidity, it remains challenging to demonstrate an impact when active transmission is ongoing. Further work is needed to define measurable morbidity and what proxy markers may be suitable to define schistosomiasis control in children. In addition, further exploration of the possible interrelationship between schistosomiasis and malaria in high transmission zone needs to be considered in future studies.

## Supplementary information

**Additional file 1 **These are details of the raw data for the study participants (*n* = 295) used in the analyses of results presented in the current paper.

## Data Availability

All data supporting our findings are already contained within this manuscript or are provided in Additional file 1.

## Abbreviations

ALB: Albendazole
EF: Emotional Functioning
EPG: Eggs per gram
HM: Hepatomegaly
IRB: Institutional Review Board
KEMRI: Kenya Medical Research Institute
MDA: Mass drug administration
PBT: Portal branch thickening
PedsQoL: Pediatrics Quality of Life
PF: Physical Functioning
PVD: Portal vein diameter
PZQ: Praziquantel
SchF: School Functioning
SERU: Scientific and Ethical Review Unit
SF: Social Functioning
SM: Splenomegaly
SPSS: Statistical Package for Social Sciences
WHA: World Health Assembly
WHO: World Health Organization
QoL: Quality of Life
